# A Rare Case of Poisoning Due to γ-Benzene Hexachloride and Cetrimide (Labscab) Lotion Ingestion

**DOI:** 10.7759/cureus.91495

**Published:** 2025-09-02

**Authors:** Mohammad Abdurrahman Khan, Pratibha Dwivedi, Manisha Verma, Anoop Kumar Verma

**Affiliations:** 1 Department of Forensic Medicine and Toxicology, Hind Institute of Medical Sciences, Lucknow, IND; 2 Department of Anatomy, Hind Institute of Medical Sciences, Lucknow, IND; 3 Department of Dentistry, Hind Institute of Medical Sciences, Lucknow, IND; 4 Department of Forensic Medicine and Toxicology, King George's Medical University, Lucknow, IND

**Keywords:** antiseptics, benzene hexachloride, cetrimide, insecticide, labscab, poisoning

## Abstract

γ-Benzene hexachloride and cetrimide (Labscab) lotion is commonly used for the treatment of scabies and pediculosis. It contains a combination of γ-benzene hexachloride (1% w/v), an insecticide, and cetrimide (0.1% w/v), a widely used antiseptic in both household and hospital settings. Accidental or intentional ingestion can lead to nausea, vomiting, esophageal injury, and tissue necrosis. We report the case of a 15-year-old unmarried female who ingested Labscab lotion. She presented with mild epigastric pain and nausea, without episodes of vomiting. Management included gastric lavage with 1000 mL of 0.9% normal saline, prophylactic antiepileptic therapy, and supportive care. The patient recovered without complications. This case highlights the importance of early hospitalization, prompt gastric lavage, supportive management, and prophylactic anticonvulsant therapy to prevent seizures and facilitate recovery. Reports of Labscab poisoning and its management remain scarce in the literature. Here, we describe a rare case of Labscab ingestion in an adolescent female and outline its successful management.

## Introduction

Benzene hexachloride (BHC) is formed through the light-induced addition of chlorine to a benzene ring. It was first produced by Michael Faraday in 1825, and its insecticidal properties were identified in 1944. BHC has eight stereoisomers, but the gamma isomer (γ-benzene hexachloride) exhibits the strongest insecticidal activity. The commercial name of γ-benzene hexachloride is lindane [1]. Commonly referred to as gammaxene, it has been widely used as an insecticide in both urban and rural households [2]. Since 1950, BHC has also been employed in the treatment of pediculosis and scabies (introduced as a scabicide). Its mechanism of action involves absorption into the chitin layer of insects, where it antagonizes the inhibitory effect of γ-aminobutyric acid channels via benzodiazepine receptors, ultimately leading to neuronal hyperexcitability, paralysis, and death [3].

In humans, neurotoxicity is the most frequently reported adverse effect of BHC. Being lipid-soluble, it readily penetrates the lipid-rich white matter of the brain. According to the FDA, approximately 70% of BHC-related adverse effects are neurotoxic, manifesting as disorientation, ataxia, tremors, seizures, and, in severe cases, death [3,4].

Cetrimide, a common component of household and hospital antiseptics, is used for skin antisepsis, hair shampooing, and the sterilization of instruments. It is generally regarded as safe; however, as a tertiary ammonium compound, ingestion can result in nausea, vomiting, esophageal damage, and necrosis. Aspiration of cetrimide solution may lead to acute respiratory distress syndrome (ARDS) [5,6].

Labscab lotion, indicated for the treatment of scabies and pediculosis (head lice), is a combination of γ-benzene hexachloride (1% w/v) and cetrimide (0.1% w/v). Reports of poisoning involving this combination and its management are exceedingly rare in the literature. Here, we describe a rare case of Labscab poisoning in a 15-year-old female and discuss its management.

## Case presentation

**Figure 1:**
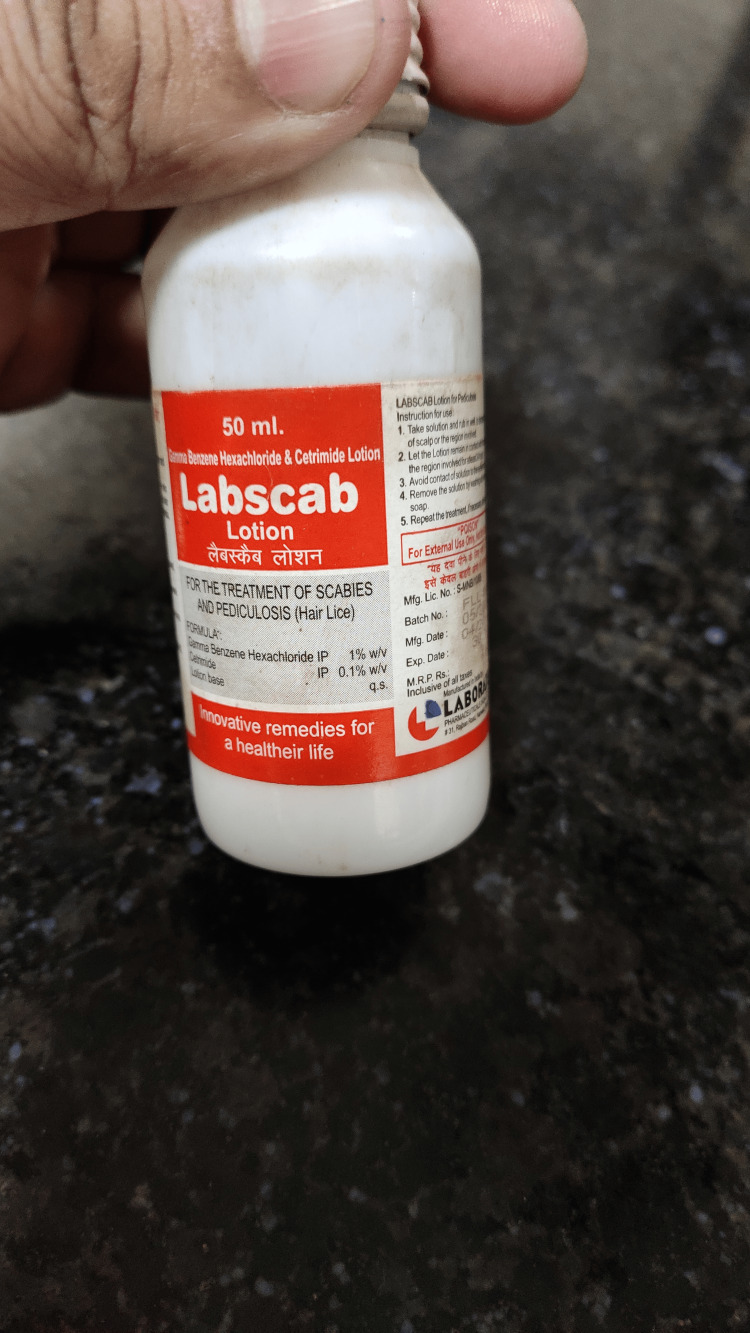
Labscab lotion bottle

A 15-year-old unmarried female presented to the Toxicology Unit of the Critical Care Medicine Department at the Trauma Centre, King George’s Medical University, Lucknow. According to the history provided by the patient, she had been apparently well until two hours prior to admission, when she ingested approximately 30 mL of Labscab lotion (γ-benzene hexachloride and cetrimide) following a quarrel with her elder sister. She also brought the empty bottle of Labscab lotion with her (Figure 1). At presentation, the patient reported mild epigastric pain and nausea but no episodes of vomiting. She had no history of similar events in the past and no significant family, medical, surgical, or menstrual history. She also denied alcohol consumption and smoking.

At the time of admission, the respiratory rate was 24 breaths/min, heart rate 91 beats/min, blood pressure 112/50 mmHg, temperature 98.4°F, and urine output 90 mL/hour. The Glasgow Coma Scale score was E4V5M6. Both pupils were normal and reactive to light. Oral examination revealed no abnormalities or lesions. The chest was bilaterally clear, although the patient had mild breathlessness, and heart sounds (S1 and S2) were normal. No focal neurological deficit or signs of meningeal irritation were noted. All reflexes were normal, with intact sensory and motor functions. The ECG was normal, while no abnormalities were detected on venous blood gas analysis.

**Figure 2:**
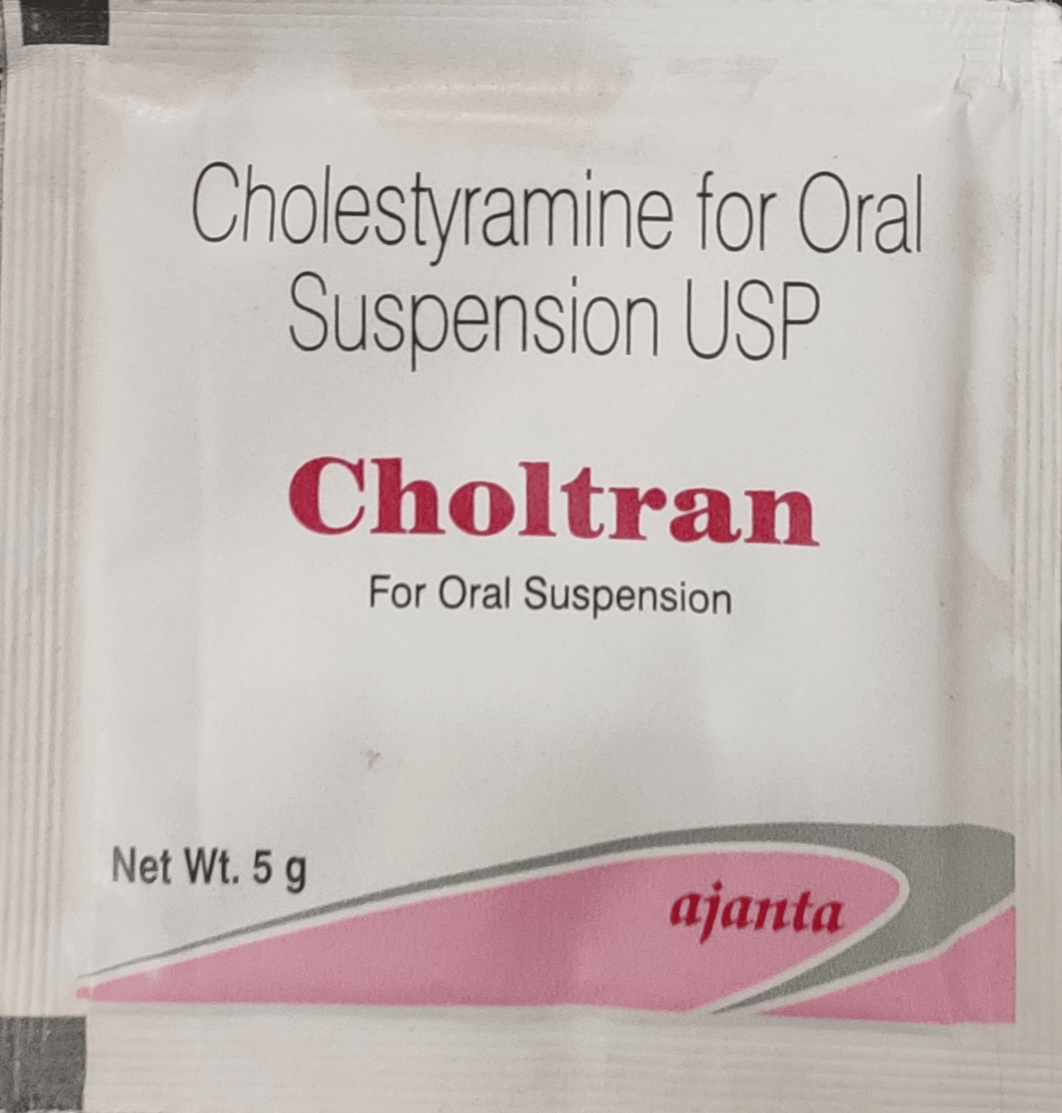
Cholestyramine sachet

For management, the patient underwent gastric lavage with 1000 mL of 0.9% saline. She was initially treated with pantoprazole 40 mg intravenously, levipil (levetiracetam) 750 mg as a loading dose followed by 500 mg twice daily, injection metoclopramide (perinorm) 10 mg intravenously as needed, cholestyramine 5 g powder orally as a stat dose (Figure 2) followed by 8 g in two divided doses, and injection vitamin B-complex (Optineuron) 1 ampule stat. The patient subsequently stabilized, maintained oxygen saturation on room air, and tolerated oral feeds.

Hematological examination on the day of admission revealed a total leukocyte count of 24,500 cells/mm³, which was markedly elevated. The differential leukocyte count showed neutrophils at 88%, lymphocytes at 10%, eosinophils at 1%, monocytes at 1%, and basophils at 0%. Renal and liver function tests were normal, while PT/INR, chest X-ray, ECG, and other investigations were also within normal limits (Table 1).

**Table 1:** Hematological parameters on day 1 and day 2 RBCs: red blood cells, MCV: mean corpuscular volume, fL: femtoliter, MCH: mean corpuscular hemoglobin, pg: picogram, MCHC: mean corpuscular hemoglobin concentration, RDW: red cell distribution width, HCT: hematocrit, PCT: plateletcrit, SGOT: serum glutamic-oxaloacetic transaminase, SGPT: serum glutamic-pyruvic transaminase, Na+: sodium, K+: potassium, Ca++: calcium

Hematology	Day 1	Day 2	Biological reference interval
Hemoglobin	13.52 g/dl	12.29 g/dl	12-15
Total leucocyte count	24500 cells/mm^3^	7705 cells/mm^3^	4000-11000
Differential leucocyte count	88, 10, 01, 01 00	75, 20, 02, 03 00	40-80, 20-40, 1-6, 1.0-5.0, 0.0-2.0
Platelets count	1.52 Lac cells/mm^3^	1.20 Lac cells/mm^3^	1.5-4.5
Total RBCs	4.152 million cells/mm^3^	3.868 million cells/mm^3^	3.8-4.8
MCV	100.1 fL	99.72 fL	80-100
MCH	32.56 pg	31.77 pg	27-32
MCHC	32.53 g/dl	31.86 g/dl	32-35
RDW	14.04%	14.09%	11.5-14.5
HCT	41.57%	38.58%	36-46
PCT	0.23%	0.1988%	0.10-0.28
Serum urea	25.2 mg/dl	27.4 mg/dl	10-45
Serum creatinine	0.83 mg/dl	0.83 mg/dl	0.5-1.4
Serum bilirubin total	1.24 mg/dl	1.26 mg/dl	0.3-1.4
Serum bilirubin direct	0.89 mg/dl	0.4 mg/dl	0-0.4
SGOT	27.2 IU/L	28.0 IU/L	0-40
SGPT	17.4 IU/L	16 IU/L	0-40
Serum alkaline phosphatase	197.2 IU/L	200 IU/L	50-240
Na+	143.6 mg/dl	144.6 mg/dl	136-145
K^+^	4.7 mg/dl	4.9 mg/dl	3.50-5.10
Ca++	9.7 mg/dl	9.6 mg/dl	8.5-10.8

On day 2, all hematological parameters returned to normal (Table 1). A psychiatric evaluation was performed. On day 3, the patient was discharged. At the time of discharge, her respiratory rate was 24/min, heart rate was 70/min, blood pressure was 122/86 mmHg, and urine output was 100 mL/hour. No treatment was prescribed at discharge. The patient was advised to return for follow-up after seven days. At follow-up, she was cheerful, well oriented to time, place, and person, and assured that she would not repeat such an act in the future.

## Discussion

Poisoning due to lindane and cetrimide is rarely reported in the literature. Such poisoning is an emergency and requires prompt management of the case since there is a risk of seizure. Lindane is the most potent toxic gamma isomer of BHC. It acts by suppressing the GABA-mediated synaptic inhibition. Most poisoning from lindane occurs through dermal contact. Poisoning with lindane due to ingestion is uncommon [7]. Lindane, if ingested, can cross the blood-brain barrier and be deposited in the white matter of the brain, potentially interfering with the metabolism of ammonia. It is associated with side effects such as nausea, vomiting, and signs of CNS overactivity such as convulsions and hyperexcitability. Other side effects associated with lindane are skin irritation, muscular cramps and dizziness, hyperpyrexia, hyperglycemia, pulmonary edema, and even death [8]. On ingestion, cetrimide produces nausea and vomiting and even esophageal damage and necrosis [6].

In our case, the patient had ingested approximately 50 mL of Labscab lotion, which contained lindane and cetrimide. Since there was no antidote for lindane and cetrimide, all the management was symptomatic. In our case, the patient complained of mild epigastric pain, nausea, and breathlessness. In our case, convulsions and CNS hyperexcitability were absent. Rapid and immediate gastric lavage, combined with symptomatic and supportive treatment, alleviated the patient's symptoms. Despite no convulsion in our patient, prophylactic treatment with levetiracetam (Levipil) 750 mg was given to the patient since there was a risk of convulsion associated with lindane. Gandhi et al. reported a case of a one-year-old child whose mother was suffering from scabies and was prescribed medication, lindane. Her child ingested lindane, and after two hours, the child began to experience episodes of seizure [9]. One study reported the case of a 25-year-old male who presented with the complaint of throat pain and vomiting following a suicidal attempt by ingesting gammaxene, and later, after 32 hours, he developed seizures. An MRI of the patient's brain revealed thrombosis of the superior sagittal vein, and later the patient was managed with phenytoin [10].

Paul et al. reported a suicidal ingestion of a 30-year-old farmer with BHC who developed both acute renal failure and acute fulminant liver failure. Renal failure recovered after hemodialysis, whereas liver function was gradually restored over time [2]. Liver and kidney failure were not seen in our case, though at the time of admission, the serum bilirubin was slightly raised, which became normalized on the next day of admission.

One study reported that lindane induced rhabdomyolysis, resulting in acute kidney injury. The patient required hemodialysis at the end of the first week, for the next week on alternate days, and supportive care. Urine output gradually improved, and creatinine was on a declining trend. The patient's muscular tenderness gradually improved as the uremic symptoms subsided and creatinine levels returned to normal [11]. In our case, early hospital arrival and ICU admission with prompt gastric lavage and early supportive care prevent kidney injury, and prophylactic anticonvulsant treatment (levetiracetam in the form of injection and cholestyramine as an adsorbent) prevents CNS complications, including convulsions.

Singh et al. reported a case of a 35-year-old pregnant chronic alcoholic female who ingested cetrimide (Savlon) and presented with dyspnoea, severe abdominal pain in the periumbilical region associated with many episodes of vomiting, and altered sensorium. The change in the patient was due to aspiration and hypoxia. Later, the patient expired following cardiac arrest [5]. Chan reported similar poisoning due to cetrimide in seven patients having symptoms of nausea, vomiting, and pain in the throat and abdomen. Out of seven, one patient became comatose and developed aspiration pneumonia and ARDS. His study suggested that symptoms of ARDS associated with cetrimide poisoning were due to aspiration [6]. In our study, only mild symptoms appeared; there was no aspiration pneumonia or ARDS since early gastric lavage and symptomatic treatment were started.

## Conclusions

The study concluded that the ingested poisoning due to Labscab lotion, which contains γ-benzene hexachloride and cetrimide, causes nausea, vomiting, abdominal pain, and neurotoxicity such as neuronal hyperexcitability and seizures. Hence, early hospitalization, prompt gastric lavage, and early supportive care, along with prophylactic anticonvulsive therapy, are needed not only to prevent the complications of seizures but also for faster recovery.
